# Stigma Toward Bariatric Surgery in the Netherlands, France, and the United Kingdom: Protocol for a Cross-cultural Mixed Methods Study

**DOI:** 10.2196/36753

**Published:** 2022-04-28

**Authors:** Franshelis K Garcia, Kirsten T Verkooijen, Esther J Veen, Bob C Mulder, Maria A Koelen, Eric J Hazebroek

**Affiliations:** 1 Health and Society, Department of Social Sciences, Wageningen University Wageningen Netherlands; 2 Rural Sociology, Department of Social Sciences, Wageningen University Wageningen Netherlands; 3 Almere University of Applied Sciences Almere Netherlands; 4 Strategic Communication, Department of Social Sciences, Wageningen University Wageningen Netherlands; 5 Human Nutrition and Health, Department of Agrotechnology and Food Sciences, Wageningen University Wageningen Netherlands; 6 Department of Bariatric Surgery, Vitalys Rijnstate Hospital Arnhem Netherlands

**Keywords:** bariatric surgery, obesity surgery, weight loss surgery, stigma, cross-cultural study, France, the Netherlands, the United Kingdom

## Abstract

**Background:**

Bariatric surgery is an effective procedure for the treatment of obesity. Despite this, only 0.1% to 2% of eligible individuals undergo surgery worldwide. The stigma surrounding surgery might be a reason for this. Thus far, no research has systematically studied the nature and implications of bariatric surgery stigma. The limited studies on bariatric surgery stigma are often conducted from the perspective of the public or health care professions and either use small and nonrepresentative samples or fail to capture the full essence and implications of the stigma altogether, including attitudes toward patients and perpetrators of the stigma. In addition, studies from patients’ perspectives are limited and tend to address bariatric surgery stigma superficially or implicitly. Finally, the extent to which cultural factors shape and facilitate this stigma and the experiences of patients have not yet been researched.

**Objective:**

This study aimed to explore the perceptions, experiences, and consequences of bariatric surgery stigma from the perspective of the public, health care professionals, and patients before and after bariatric surgery. Furthermore, although the concept of stigma is universal, every society has specific cultural norms and values that define acceptable attributes and behaviors for its members. Therefore, this study also aimed to explore the extent to which cultural factors influence bariatric surgery stigma by comparing the Netherlands, France, and the United Kingdom.

**Methods:**

This paper describes the protocol for a multiphase mixed methods research design. In the first part, we will conduct a scoping review to determine the current knowledge on bariatric surgery stigma and identify knowledge gaps. In the second part, semistructured interviews among patients before and after bariatric surgery will be conducted to explore their experiences and consequences of bariatric surgery stigma. In the third part, surveys will be conducted among both the public and health care professionals to determine the prevalence, nature, and impact of bariatric surgery stigma. Surveys and interviews will be conducted in the Netherlands, France, and the United Kingdom. Finally, data integration will be conducted at the interpretation and reporting levels.

**Results:**

The study began in September 2020 and will continue through September 2025. With the results of the review, we will create an overview of the current knowledge regarding bariatric surgery stigma from patients’ perspectives. Qualitative data will provide insights into patients’ experiences with bariatric surgery stigma. Quantitative data will provide information related to the prevalence and nature of bariatric surgery stigma from the perspective of the public and health care professionals. Both qualitative and quantitative data will be compared for each country.

**Conclusions:**

The findings from this study will lead to new insights that can be used to develop strategies to reduce bariatric surgery stigma and improve access, use, and outcomes of bariatric surgery.

**International Registered Report Identifier (IRRID):**

PRR1-10.2196/36753

## Introduction

### Background

Obesity (BMI ≥30 kg/m^2^) has become a major health issue worldwide. People with obesity are at risk for several conditions, including cardiovascular disease, diabetes, hypertension, infertility, arthritis, and certain types of cancer [1]. More severe forms of obesity are associated with lower quality of life and higher chances of morbidity and mortality [2]. For people with severe obesity (BMI ≥40 kg/m^2^ or BMI ≥35 kg/m^2^ with ≥1 obesity-related condition), bariatric surgery is considered the only effective treatment when diet, exercise, or pharmacological interventions do not result in sufficient or permanent weight loss [3-5]. There are numerous types of bariatric surgery; however, essentially, all involve the surgical alteration of the stomach or intestines to restrict the intake or absorption of food [6]. People with severe obesity undergoing bariatric surgery lose an average of approximately 45% to 58% of their excess body weight, depending on the type of procedure performed, and maintain this weight loss in the long term [7]. In addition to sustained weight loss, there is increasing evidence that bariatric surgery leads to improvement in obesity-related comorbidities such as type 2 diabetes and hypertension [3-5,8], thereby reducing the risk of mortality [5].

Despite the effectiveness of bariatric surgery, worldwide, only 0.1% to 2% of people with obesity who are eligible for bariatric surgery undergo surgery [9]. Underuse of bariatric surgery is largely related to unequal access to care, misconceptions about the safety and efficacy of surgery, and cultural and social bias and stigma [10,11]. Similar to obesity, bariatric surgery is highly stigmatized, with surgery being viewed as a *last resort* and an *easy way out* method to lose weight [11]. In addition, people with obesity who choose or have bariatric surgery are stigmatized and perceived by others as *lazy*, *sloppy*, *less competent*, and *lacking in self-discipline* compared with people with obesity who lose weight through diet and exercise [12-17]. The negative attitudes toward the procedure and patients of bariatric surgery stem from the assumptions about personal responsibility in obesity and the misconceptions about surgery being an *easy fix* and *low-effort* method of losing weight [9,13,16].

There are numerous potential consequences of the prevailing negative attitudes toward bariatric surgery and patients [11,18]. For example, people who are eligible for surgery may not be referred by health care professionals because of the stigma surrounding the procedure [19,20]. People with obesity who internalize and agree with stigmatizing beliefs may be discouraged from considering surgery [12,15,21] out of fear of being judged by others as taking the *easy way out*. This fear of judgment may also withhold those who are considering or have undergone surgery from disclosing their surgery status [11,13,15,22-24], which can limit opportunities for social support. After surgery, stigma may hamper patients’ ability to adhere to necessary postsurgical dietary and behavioral recommendations [25,26] and therefore decrease surgery success.

Currently, numerous studies have documented obesity stigma (stigma toward people with obesity) in many countries and diverse populations [14,16,18,27-35]; however, research on bariatric surgery stigma is limited and often conducted from the perspective of the public [12,14,16,17,29,30,32,36-42] and health care professionals [20,36,43-46]. These studies provide valuable insights into the prevalence and nature of bariatric surgery stigma. However, they have several limitations. First, many studies on bariatric surgery stigma have focused on attitudes toward the procedure itself rather than on attitudes and behaviors toward people who are considering or have undergone bariatric surgery. Stigma is a term used to describe the negative way in which we think about, feel, and act toward individuals who are different from us, as these individuals possess certain socially unacceptable characteristics or attributes [47]. Therefore, to fully capture the prevalence and nature of bariatric surgery stigma, research needs to address not only the stigma attached to the procedure but also the stigma attached to the person who is considering or has undergone bariatric surgery. Thus far, only 4 studies have investigated attitudes toward patients of bariatric surgery, all of which were conducted among the public. However, all of these 4 studies included small sample sizes [14,16,17,39], and 3 used nonrepresentative samples (eg, undergraduate or psychology students) [14,16,39], limiting their generalizability.

Second, although the stigma toward the procedure has been researched extensively, how this stigma is conceptualized and measured differs significantly between studies. For instance, most studies addressing stigma toward bariatric surgery measure stigma in terms of *belief in public versus private funding* [12,41,42], *willingness to recommend or undergo surgery* [20,29,32,36-38,43,45,46], *perception of surgery as medical or cosmetic* [12,37], and *agreement with easy way out* [12,20,40]. This lack of clear conceptualization and the various ways of measuring stigma make it difficult to compare the results of different studies and hinder the accumulation of knowledge about bariatric surgery stigma and its consequences. In addition, many studies that claim to measure stigma toward bariatric surgery actually measure individual-level factors that trigger the occurrence and perpetuation of the stigma, such as knowledge about and perceived effectiveness of surgery [20,29,30,32,37,45,46], rather than the actual stigma. This is often the case for studies among health care professionals. Although these studies provide valuable information regarding the factors that cause and perpetuate the stigma, important aspects of the stigma, stereotypes, and prejudice remain underexamined. Understanding what stereotypes and prejudices are associated with bariatric surgery, and thus how bariatric surgery is framed, is important as it influences how people think about, feel, and act toward patients of bariatric surgery. In addition, acquiring this knowledge is a key step in intervening and tackling negative stereotypes, prejudices, and behaviors toward patients of bariatric surgery.

Third, most research into bariatric surgery stigma thus far has focused on the stigma from the perspective of stigmatizers. Although these studies among the public [12,14,16,17,29,30, 32,36-42] and health care professionals [20,36,43-46] provide evidence that stigma toward patients of bariatric surgery exists, they provide no knowledge regarding the actual experiences of patients with the stigma and the consequences thereof. The importance of understanding the perspectives of patients of bariatric surgery is reflected in the number of studies published over the past years [21,48,49]. These studies highlight the impact of bariatric surgery on many different aspects of patients’ lives and the challenges patients face after surgery. However, they provide limited information regarding patients’ experiences with bariatric surgery stigma. Existing studies among patients of bariatric surgery mostly focus on weight stigma and the implications thereof and tend to address bariatric surgery stigma superficially or implicitly, presenting a limited snapshot of the actual stigmatizing experiences of patients [21,48]. Studies from patients’ perspectives are vital to obtaining a better understanding of the extent to which patients of bariatric surgery perceive, experience, and internalize bariatric surgery stigma; how they respond to this stigma; and how this stigma affects patients’ everyday lives [13,24].

Finally, existing studies on bariatric surgery stigma have documented the presence of this stigma in several parts of the world, varying from Western countries (eg, Germany, the United Kingdom, the United States, and Australia) [29,32,36] to less westernized countries (eg, Saudi Arabia and China) [37,44]. Despite this, we know relatively little about whether and to what extent cultural factors such as social, beauty, and gendered norms shape people’s attitudes, behaviors, and experiences of stigma. Stigma is a social construction influenced by cultural, historical, and situational factors and is constantly evolving within social interactions, norms, context, and values [50]. Individual characteristics may be stigmatized at one historical moment but not at another or in one specific place or context but not in another within the same period [51]. Hence, although the concept of stigma is universal, every society has specific cultural norms, values, and structures that define acceptable attributes and behaviors for its members. Consequently, the perception of what constitutes stigma may vary from one society to another [34,51]. Cultures emphasizing fashion, luxury, and a slender body as a reflection of the ideal feminine beauty, such as in France, might promote obesity stigmatization, particularly among women [52]. However, it is unclear whether these norms would also facilitate the stigmatization and experiences of patients of bariatric surgery. This illustrates the fundamentally social nature of stigma and the need for research into the cultural factors that shape and facilitate the occurrence of bariatric surgery stigma and the experiences of patients.

### Aims and Objectives

Currently, no studies have systematically explored bariatric surgery stigma. Consequently, our understanding of the prevalence, nature, and implications of bariatric surgery stigma remains limited. Many studies aiming to increase the understanding of bariatric surgery stigma have been conducted from the perspective of the public or health care professionals and tend to focus mostly on the stigma associated with the procedure rather than the stigma toward patients. Studies that investigate public attitudes toward patients of bariatric surgery use small and nonrepresentative samples and different ways of conceptualizing and measuring stigma, limiting the generalizability of and comparability between studies. Moreover, many studies from the perspective of the public and health care professionals actually measure factors that cause or perpetuate bariatric surgery stigma. Therefore, our understanding of what the stigma related to bariatric surgery entails, both for the procedure and toward patients, remains limited. In addition, our knowledge of patients’ experiences is limited as many studies among patients of bariatric surgery tend to address it implicitly. Finally, the extent to which cultural factors shape and facilitate bariatric surgery stigma and the experiences of patients has not yet been researched.

Given the limited knowledge and lack of systematic research on the prevalence, nature, and consequences of bariatric surgery stigma, the overall aim of this study is to explore the perceptions, experiences, and consequences of bariatric surgery stigma from the perspective of (1) people with obesity who are considering or about to undergo surgery (patients before bariatric surgery), (2) people who have undergone surgery (patients after bariatric surgery), (3) the general public, and (4) health care professionals. In addition, this study aims to explore the extent to which bariatric surgery stigma is shaped by cultural factors by comparing three European countries: the Netherlands, France, and the United Kingdom. These countries were chosen as they are geographically and socioeconomically close but differ significantly in their views toward and approaches to tackling obesity. In addition, to date, no research has been conducted on the prevalence, nature, and consequences of bariatric surgery stigma in the Netherlands and France.

The objectives of the research are as follows:

1. To determine the current knowledge on stigma toward bariatric surgery and its consequences from the perspective of patients of bariatric surgery and identify possible knowledge gaps
2. To explore the experiences and consequences of bariatric surgery stigma from the perspective of patients of bariatric surgery
3. To determine the prevalence and nature of bariatric surgery stigma in the general public and among health care professionals
4. To assess the extent to which bariatric surgery stigma is culture dependent by comparing the Netherlands, France, and the United Kingdom.

## Methods

### Theoretical Framework

The theoretical framework guiding this study is the Health Stigma and Discrimination Framework (HSDF) developed by Stangl et al [53] (Multimedia Appendix 1). The HSDF is a multilevel theoretical framework that “articulates the stigmatization process as it unfolds across the socio-ecological spectrum in the context of health” [53]. This framework comprises several domains, including drivers and facilitators of stigma, stigma markings, and stigma manifestations, which, in turn, influence the main outcomes (eg, coping, adherence to treatment, and accessibility to quality health care) and, ultimately, health and quality of life among the stigmatized group. Drivers and facilitators of stigma determine the occurrence and perpetuation of stigma (stigma markings). Drivers (individual-level factors) are inherently negative and include, for example, blame and attributions of responsibility and control. Drivers of stigma may originate from, for example, lack of knowledge. Facilitators (social-level factors), on the other hand, may be positive or negative and include cultural and gender norms and (institutional) laws, policies, and regulations.

Within this framework, stigma can manifest itself in terms of stigma practices and stigma experiences. Stigma practices are defined from the perspective of *those who stigmatize* and include stereotypes (ie, labels and beliefs about the characteristics of a group), prejudice (ie, endorsement of beliefs and negative evaluations), and stigmatizing behavior and discrimination (ie, unfair treatment and social rejection) [53]. Stigma experiences are defined from the perspective of people who are stigmatized and include actual experiences of stigmatizing acts and discrimination (experienced stigma; eg, negative comments from friends), perceptions of stigma (perceived stigma; eg, “People think I cheated by having surgery”), anticipation or expectation of discrimination or social rejection if one’s condition becomes known (anticipated stigma; eg, “I’m afraid of how my friends would react if they found out I had surgery”), and the internalization of stigma (internalized stigma; eg, “It was the surgery, not me that made me lose weight and I felt ashamed”).

We will apply the HSDF to data analysis (ie, the development of data extraction forms and coding categories; details are provided in the following sections). Furthermore, we will use this framework to highlight how and at what levels bariatric surgery stigma operates and affects individuals who are considering or have undergone surgery. In addition, the framework will also be used to highlight the similarities and differences between countries. Finally, we will use the HSDF to help identify which multiple-level interventions are needed to meaningfully intervene and combat bariatric surgery stigma.

### Study Design

**Figure 1:**
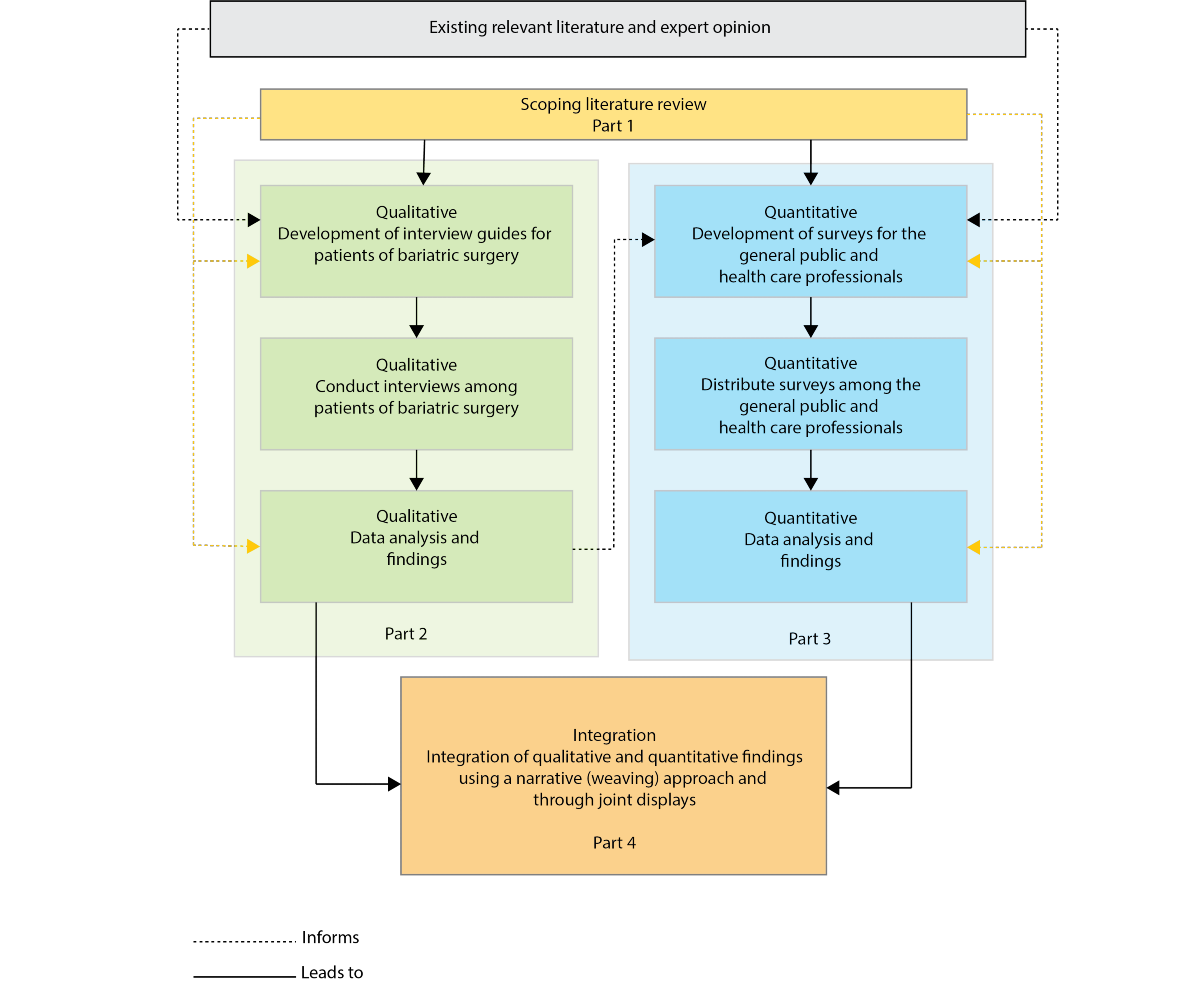
Multiphase mixed methods research design comprising 4 parts.

This study uses a multiphase mixed methods research design with an exploratory sequential and convergent component (Figure 1) [54,55]. This type of research design examines a topic or problem through a series of phases or separate studies. Although each phase stands on its own (convergent component), these phases are connected sequentially (exploratory sequential components). This study will be conducted in 4 parts, each addressing the corresponding objectives. The first part addresses research *objective 1* through a literature review. The results of this part will also be used to inform the development of interview guides, surveys, and analyses. The second part, the qualitative phase, is directed toward achieving research *objectives 2 and 4*. In this part, patients of bariatric surgery will be interviewed regarding their experiences with bariatric surgery stigma and the consequences of this stigma before and after surgery. We will use the findings from baseline qualitative interviews to further inform the development of the surveys (eg, identify additional domains, language use, and wording of items) [54]. The third part, the quantitative phase, addresses research *objectives 3 and 4*. We will conduct surveys among the public and health care professionals to determine the prevalence and nature of bariatric surgery stigma. Although the findings of the interviews will be used to inform the development of the surveys, each method will address a specific research objective. Consequently, both qualitative and quantitative data collection can occur concurrently [54,55]. The findings of the interviews and surveys, each analyzed and synthesized individually, will be combined afterward to create a better and holistic understanding of the research topic and draw valid conclusions (part 4) [54,55]. The research, methods, and activities for each part are explained in more detail in the following sections.

### Study Setting and Study Population

We will collect data from the Netherlands, France, and the United Kingdom. Although these countries are geographically and socioeconomically close [56], the way their health care systems are organized [57-59] and their views of and approaches to tackle obesity [60-65], as well as their organization of, access to, and rates of bariatric surgery, differ significantly [65].

The study population comprises 4 different groups:

1. Patients before bariatric surgery, comprising people with severe obesity (BMI ≥40 kg/m^2^ or BMI ≥35 kg/m^2^ with ≥1 obesity-related condition) considering, awaiting, scheduled, or about to undergo bariatric surgery
2. Patients after bariatric surgery, comprising people who have undergone bariatric surgery
3. The general public
4. Health care professionals involved in the bariatric surgery care pathway [60-65]

### Part 1: Literature Review

To determine the current knowledge on stigma toward bariatric surgery from the perspective of patients of bariatric surgery and identify knowledge gaps (objective 1), we will conduct a scoping literature review adhering to the PRISMA-ScR (Preferred Reporting Items for Systematic Reviews and Meta-Analyses Extension for Scoping Reviews) [66]. A total of four electronic databases will be searched: PubMed, Web of Science, PsycINFO, and MEDLINE. The search terms will include 2 main keywords, *stigma* and *bariatric surgery*, and will be expanded with synonyms and Medical Subject Heading terms. Both qualitative and quantitative studies addressing the experiences and perceptions of stigma for patients before and after bariatric surgery will be considered for inclusion. A data extraction form developed iteratively using the HSDF and piloted by a minimum of 2 reviewers will be used to extract data from all relevant papers and include information on study characteristics (eg, author, year of publication, and country), sample characteristics (eg, gender, age, type, and time of surgery), and stigma-related findings.

### Part 2: Qualitative Phase—Interviews

#### Data Collection: Semistructured Interviews

Semistructured interviews will be conducted to explore the experiences and consequences of bariatric surgery (objective 2) from the perspective of patients before and after bariatric surgery and assess the extent to which this experienced stigma is culture dependent (objective 4). We developed a preliminary interview guide based on relevant literature [23,67], the results from the scoping literature review, and consultations with experts in the field of bariatric surgery (eg, bariatric surgeons). Iterative changes to the interview guides will be made if needed. The interview guides will be initially developed in English and forward and backward translated into the Dutch and French languages by independent translators. For both patients before and after bariatric surgery, the interview guides will broadly explore the following themes: (1) personal, public, and health care professionals’ views of bariatric surgery; (2) motivation and decision-making for bariatric surgery; (3) disclosure of bariatric surgery and stigma experiences; and (4) expected changes after surgery or changes since surgery.

#### Sampling and Recruitment

Participants aged ≥18 years considering, awaiting, scheduled, about to undergo, or have undergone bariatric surgery and living in the Netherlands, France, or the United Kingdom will be recruited for interviews. The main criterion for patients before bariatric surgery is that participants need to fulfill the BMI criteria to undergo bariatric surgery (BMI ≥40 kg/m^2^ or BMI ≥35 kg/m^2^ with ≥1 obesity-related condition). Purposive sampling will be used to recruit patients both before and after bariatric surgery. Purposive sampling is a cost-efficient sampling method that is useful for qualitative research as it allows the researcher to intentionally select participants according to predetermined criteria relevant to a particular research objective [68-70]. To provide a wide range of perspectives, we aim to include patients from different age groups, genders, and socioeconomic statuses. Therefore, participants for the interview will be recruited via health care professionals, (digital) posters and (digital) flyers at clinics and hospitals, social media (eg, web-based community and associations on Facebook, Twitter, or Instagram), forums, and in person during events for people who are about to or have undergone bariatric surgery. Participants who express interest will be screened for eligibility, provided with information about the study, and scheduled for an interview at their desired location. We will interview the participants only after they have provided their informed consent. On the basis of previous studies, we anticipate that the interviews would last between 30 and 90 minutes [71-74].

Data collection will stop when data saturation is reached. Data saturation occurs when no new information is obtained [69,70], typically by 12 interviews in qualitative studies. To ensure data saturation, we will conduct a minimum of 24 interviews (12 before and 12 after bariatric surgery) in each country.

### Part 3: Quantitative Phase—Surveys

#### Data Collection: Surveys

Surveys will be used to determine the prevalence and nature of bariatric surgery stigma (objective 3) from the perspective of the general public and health care professionals and assess the extent to which this stigma is context and culture dependent (objective 4). We developed a preliminary version of the survey based on existing relevant literature [13,30,32,36-38,43-46, 75-78], expert opinions, and the preliminary results of the scoping literature review. The survey is segmented into several parts: the first part assesses whether respondents meet the eligibility criteria for the survey and includes 3 questions that can be answered with either *yes* or *no*: “Are you 18 years or older?”; “Do you live in the Netherlands, France, or the United Kingdom?”; and “Are you able to read and understand English, Dutch, or French?” Only respondents who answer *yes* to all 3 questions and provide informed consent will be allowed to continue with the remainder of the survey. The second part of the survey collects information on knowledge and attitudes toward obesity and its treatment. The third part collects information on knowledge and attitudes toward bariatric surgery. We will measure both positive and negative attitudes toward bariatric surgery. As recommended by Rattray and Jones [79], to engage respondents, the final part of the survey collects demographic information such as age, gender, self-reported height and weight, country of residence, ethnicity, educational level, marital status, and occupation. For health care professionals, information such as type of specialization, years of experience, and experience working with people with obesity will also be assessed. The surveys comprise both closed- and open-ended questions (eg, “Have you ever heard positive comments about people who have had bariatric surgery?...If yes—What positive comments have you heard?”).

The surveys will be developed in English, tested by experts, and translated into Dutch and French. To test the readability of the survey items and ensure conceptual and semantic equivalence, the surveys will be forward and backward translated by independent translators fluent in both English and the language of the translated survey [80]. Once conceptual and semantic equivalence is ensured, the survey will be piloted by a small group of respondents in their native language to verify whether respondents can understand and answer all the questions [81]. Once the surveys are revised and corrected for errors, if needed, the final version of the survey will be placed on the web to collect data for the study.

We will distribute a link to the web-based surveys via national and international professional organizations, national and international panels, and personal networks of health care professionals in the bariatric surgery pathway. Respondents will be asked to complete the survey within a 6-week time frame. After 3 weeks, a reminder will be sent to increase the response rate.

#### Recruitment and Sampling

We will use probability and nonprobability sampling methods to recruit members of the general public and health care professionals in each country.

Members of the general public will be recruited via paid web-based panels. The inclusion criteria for the general public are adults aged ≥18 years and living in the Netherlands, France, or the United Kingdom, who can communicate in the country’s national language (Dutch, French, or English) and give informed consent.

Health care professionals will be recruited via associations for the study of obesity (surgery; eg, the European Association for the Study of Obesity), general surgery societies (eg, the Dutch Association of Surgeons), and national and international web-based panels and hospitals in the 3 countries. For each country, we will strive to include a representative sample of health care professionals involved in the bariatric surgery care pathway, including (primary) general practitioners, practice nurses, endocrinologists, internists (internal medicine), general surgeons, bariatric surgeons, psychologists, and registered dietitians. The inclusion criteria for health care professionals are adults aged ≥18 years involved in the bariatric surgery care pathway and living in the Netherlands, France, or the United Kingdom, who can communicate in the country’s national language (Dutch, French, or English) and give informed consent.

To generalize the study findings and avoid sampling errors or biases, the minimum sample size required for the survey study among the general public is 1155 respondents (385 respondents per country). The sample size was calculated by using Raosoft Sample Size Calculator [82], assuming a CI of 95%, a margin of error of 5%, and a 50% chance of agreeing to take part in the study [82,83]. For health care professionals, the minimum sample size required was calculated assuming an unlimited population size [82]. This resulted in a minimum sample size of 1155 health care professionals (385 respondents per country). To ensure that the minimum sample size for both the general public and health care professionals is obtained, we will increase the sample size by 50%, distributing the surveys to a minimum of 1733 respondents (578 respondents per country) in the general public and a minimum of 1733 health care professionals (578 respondents per country) [83].

#### Data Analysis

##### Quantitative Data Analysis

Quantitative data analysis will be conducted using SPSS (version 27; IBM Corp). To prepare the survey for analysis, data will first be imported to SPSS and cleaned (eg, checked for missing values, recording, and computing variables). Descriptive statistics will be reported in absolute and relative values using the measures of frequency (count and percentage), central tendency (mean), and variation (SD, minimum, maximum, and range) for all quantitative data, where applicable. Inferential statistics (chi-square tests, *t* tests, and ANOVA) will be performed to analyze the differences and associations between demographic groups and countries. The significance level will be set at α=.05.

##### Qualitative Data Analysis

Interviews will be audio recorded and transcribed in full, with all personal identifiable information removed. The transcripts will then be imported into a qualitative data analysis software package (eg, Atlas.ti Scientific Software Development GmbH) for coding and analysis. The data will be analyzed using both deductive (theory-driven) and inductive (data-driven) thematic analysis approaches [84,85]. Thematic analysis is a method for identifying, analyzing, and interpreting patterns of meaning in qualitative data [84,85]. Quotations from the participants, derived from the English interviews and original Dutch and French interviews translated into English, will be used to illustrate the findings and allow readers to assess the accuracy of the analysis [86,87].

##### Data Integration of Part 2 and Part 3

According to a study by Fetter et al [54], the findings of mixed methods studies can be integrated into the design, method, and interpretation and reporting levels of research. Given the aim and multiphase design of this study, the integration will occur at 2 levels: the methods level and the interpretation and reporting level. Integration at the methods level will occur by building using findings from the scoping literature review and qualitative interviews to inform the development of the surveys. Integration at the interpretation and reporting level will be implemented by connecting the qualitative findings from part 2 of the study with the quantitative findings from part 3 of the study using a staged approach in which the results of each part (survey vs interviews) are reported in stages as the data are analyzed and published separately [54]. The findings of the quantitative and qualitative data analyses will then be brought together using a narrative (weaving) approach describing the quantitative and qualitative findings on a theme-by-theme basis using the HSDF as a guiding framework [54]. Finally, the qualitative and quantitative findings will be integrated through joint displays. Joint displays bring data together through visual means (eg, tables, graphs, figures, and matrices) and help to draw out new insights beyond the information gained from separate qualitative and quantitative findings [54].

### Ethics Approval

The Medical Research Involving Human Subjects Act does not apply to this study in the Netherlands, as confirmed in September 2021 by the Medical Research Ethics Committee of Utrecht. On January 22, 2022, the Social Science Ethical Committee of the University of Wageningen approved the qualitative part of this study. We will submit the quantitative part of the study separately for ethics approval. The study will also be submitted for review by the Medical Research Ethics Committee in France (Comité de Protection des Personne) and the United Kingdom (Research Ethics Committee).

Participation in this study is voluntary. Before the start of the survey and interviews, we will provide participants with information about the purpose and scope of the study, the length of the survey and interview, their personal rights, and data protection regulations. Data will only be collected after participants have provided informed consent. Interview participants will have the opportunity to ask questions before giving informed consent and signing the relevant consent forms. The survey participants can contact the researchers for any additional information or further questions regarding the study. All participants will be informed that they can withdraw from the interview or stop with the surveys at any time without providing any form of explanation.

Participants in both surveys and interviews will be assured of confidentiality and anonymity. Survey data will be collected anonymously using a certified web-based survey tool that complies with the European Union’s General Data Protection Regulation [88]. Interview data will be audio recorded and transcribed afterward. Once transcribed, audio recordings will be erased. All written transcriptions will be deidentified by removing names and changing any identifying data that can be linked back to the participant. All collected data will be securely stored in an encrypted password-protected folder with restricted access.

There is no major risk anticipated with filling in the survey and participating in the interviews. However, survey respondents might feel uncomfortable sharing their views on obesity and bariatric surgery. To reduce this risk, survey respondents will be informed about the potential risks and benefits of the study during the consent process so that they can choose whether to participate [89]. In addition, potential survey respondents will be informed that if they choose to participate, they can choose to skip questions or stop with the survey at any time without providing a reason [89]. The same principles apply to the interview participants. Interview participants may also feel distressed or discomfort when discussing their experiences with stigma; in this situation, participants will be asked whether they are fine and whether they would like to take a short break and continue with the remainder of the interview later on. Toward the end of the interview, we will encourage participants to contact the researcher if they experience continued discomfort or distress as a consequence of participating in the interview.

## Results

The research study started in September 2020 and will continue through September 2025. The Research and Assessment Committee from the Wageningen School of Social Sciences reviewed this study (Multimedia Appendix 2 [25,26,90,91]). We will conduct a scoping review to create an overview of the current knowledge related to the experiences and consequences of stigma for patients before and after bariatric surgery. We will also use this information to identify knowledge gaps and further inform the research methods and analyses. Data collected using quantitative methods will provide information on the prevalence and nature of bariatric surgery stigma and will be used to assess the extent to which stigma is culturally dependent. Finally, the qualitative aspect of the study is designed to generate rich information on the lived experiences of patients before and after bariatric surgery, taking into account the experiences and impact of stigma. We will compare quantitative and qualitative findings from all the 3 countries to discuss the similarities and differences between each country.

## Discussion

### Principal Findings

Obesity is becoming a major health issue in today’s society. One way of combating obesity and related diseases is through bariatric surgery. However, the widespread stigma surrounding bariatric surgery may restrict access to this procedure and influence the well-being of patients before and after bariatric surgery, in turn generating health disparities, as people who meet the criteria for bariatric surgery are often economically disadvantaged, have lower levels of education, have less access to health care, and come from racial or ethnic minorities [92,93]. Therefore, it is critically important to conduct research on the nature, experiences, and impacts of stigma. If bariatric surgery stigmatization withholds individuals with obesity from undertaking bariatric surgery or decreases the well-being of patients of bariatric surgery, it is important to alter these negative attitudes in both the general population and health care professionals.

This will be the first study to investigate bariatric surgery stigma from multiple perspectives and in multiple countries. This study is expected to lead to new knowledge and a better understanding of bariatric surgery stigma in several ways. First, although previous research predominantly focused on weight bias and obesity stigmatization, this study will lead to in-depth knowledge on bariatric surgery stigmatization as it uncovers the nature and prevalence of bariatric surgery stigma and how it is perceived and experienced by patients of bariatric surgery. Second, the research will study bariatric surgery stigma from the perspective of both the stigmatized (eg, patients before and after bariatric surgery) and the potential stigmatizer (eg, the general public and health care professionals), which will foster a more comprehensive picture of the topic. Understanding bariatric surgery stigma from the general public, health care professionals, and patients’ perspectives might help with decreasing negative attitudes. More knowledge regarding the prevalence and nature of bariatric surgery stigma and hearing about the experiences of patients of bariatric surgery may help reduce the bias and negative attitudes held by the public and health care professionals and help prepare future patients for surgery.

Third, the research will be unique in combining quantitative survey data with in-depth qualitative data to gain a full understanding of the topic. Surveys and interviews will be used as complementary methods to explore different perspectives on stigma. Although mixed methods designs generally require more time, resources, and skills in different research methods, they are valuable as they contribute to a better understanding of the problem than quantitative or qualitative research methods alone [55].

In addition, the research will compare 3 European countries (the Netherlands, France, and the United Kingdom), which will provide insights into how bariatric surgery stigmatization is constructed and maintained by its societal and cultural context. A cross-cultural study of bariatric surgery stigma will help us understand how the drivers and facilitators of stigma, stigma experiences, and discrimination are similar or different across cultures, thereby highlighting how stigma is influenced by culture. Furthermore, this study will be guided by the HSDF to help understand how bariatric surgery stigma operates at different levels and help identify what interventions are needed to combat this stigma. Finally, the topic of bariatric surgery stigma will be approached from an interdisciplinary perspective: contributions from sociology, psychology, ethics, public health, medicine, and health care will be used to document and analyze the topic.

### Limitations

In this mixed methods research, data will be collected in the national language of the country. This poses challenges for (1) the translation of surveys and interviews and (2) data analysis, as translation to a common language is time consuming and expensive, and the meaning can easily be distorted or lost in the process of translation [94]. For example, in some languages, words can have several meanings depending on the context in which they are spoken. In addition, some words or phrases (eg, *Gezellig* in Dutch and *Ras-le-bol* in French) cannot literately be translated into other languages. The accuracy and validity of the data might be compromised because of translation.

To help ensure the accuracy of the data collected and the validity of the results reported so that local meaning and cultural connotations are not lost, we will pay particular attention to the development and testing of surveys and interview topic guides and the analysis and reporting of data [80,94]. Surveys and interviews will be forward and backward translated by independent translators who have good proficiency in the language and then piloted by native speakers. To minimize the risk of misinterpretation, misunderstanding, and loss of respondents’ intended meaning and thus maintain the *conceptual equivalence*, qualitative data derived from the interviews will be analyzed in the language in which it was obtained, using an iterative coding framework with common labels. Participants interested in the study results will receive a summarized version in both English and the language of origin (Dutch or French).

For the interviews, because of time constraints and the geographical spread of hospitals and clinics that perform bariatric surgery in the Netherlands, France, and the United Kingdom, it is not possible to include participants from all regions within these countries. Consequently, not all types of patients of bariatric surgery will be represented in this study. In addition, in qualitative studies, it is often difficult to assess whether the sample is representative of the population. To ensure that a wide range of patient perspectives is included in the study, we will interview patients from different age groups, genders, and socioeconomic statuses. In addition, interviews will be conducted until data saturation is reached and, thus, no new information is discovered [69,70].

### Conclusions

This protocol outlines the rationale and design of a cross-cultural mixed methods study focused on understanding the prevalence, nature, and impact of bariatric surgery stigma from the perspective of the public, health care professionals, and patients of bariatric surgery in the Netherlands, France, and the United Kingdom. We expect the results of this study to be relevant for people with obesity who are considering bariatric surgery, people who have undergone surgery, and health care professionals involved in the care of people with obesity and patients of bariatric surgery. The findings of this study have several practical implications. It can be used to educate the general public regarding patients’ experiences, in the education of health care professionals who work with patients of bariatric surgery, by health care professionals to generate discussions with patients and raise awareness of the issue, and to develop interventions that can be used to educate patients of bariatric surgery before surgery about the potential experiences and implications of stigma to prepare them for life afterward. In turn, bariatric surgery access, use, and outcomes, including the quality of life of patients, may be improved.

## Appendix

Multimedia Appendix 1 Health Stigma and Discrimination Framework.

Multimedia Appendix 2 Peer-review report from the Wageningen School of Social Sciences.

## Abbreviations

HSDF: Health Stigma and Discrimination Framework
PRISMA-ScR: Preferred Reporting Items for Systematic Reviews and Meta-Analysis Extension for Scoping Review

## References

[1] Wolfe BM, Kvach E, Eckel RH (2016). Treatment of obesity: weight loss and bariatric surgery. Circ Res.

[2] Abdelaal M, le Roux CW, Docherty NG (2017). Morbidity and mortality associated with obesity. Ann Transl Med.

[3] Angrisani L, Santonicola A, Iovino P, Formisano G, Buchwald H, Scopinaro N (2015). Bariatric Surgery Worldwide 2013. Obes Surg.

[4] Buchwald H, Estok R, Fahrbach K, Banel D, Jensen MD, Pories WJ, Bantle JP, Sledge I (2009). Weight and type 2 diabetes after bariatric surgery: systematic review and meta-analysis. Am J Med.

[5] Chang SH, Stoll CR, Song J, Varela JE, Eagon CJ, Colditz GA (2014). The effectiveness and risks of bariatric surgery: an updated systematic review and meta-analysis, 2003-2012. JAMA Surg.

[6] Colquitt JL, Pickett K, Loveman E, Frampton GK (2014). Surgery for weight loss in adults. Cochrane Database Syst Rev.

[7] O'Brien PE, Hindle A, Brennan L, Skinner S, Burton P, Smith A, Crosthwaite G, Brown W (2019). Long-term outcomes after bariatric surgery: a systematic review and meta-analysis of weight loss at 10 or more years for all bariatric procedures and a single-centre review of 20-year outcomes after adjustable gastric banding. Obes Surg.

[8] Welbourn R, Hollyman M, Kinsman R, Dixon J, Liem R, Ottosson J, Ramos A, Våge V, Al-Sabah S, Brown W, Cohen R, Walton P, Himpens J (2019). Bariatric surgery worldwide: baseline demographic description and one-year outcomes from the fourth IFSO global registry report 2018. Obes Surg.

[9] Rubino F, Puhl RM, Cummings DE, Eckel RH, Ryan DH, Mechanick JI, Nadglowski J, Ramos Salas X, Schauer PR, Twenefour D, Apovian CM, Aronne LJ, Batterham RL, Berthoud HR, Boza C, Busetto L, Dicker D, De Groot M, Eisenberg D, Flint SW, Huang TT, Kaplan LM, Kirwan JP, Korner J, Kyle TK, Laferrère B, le Roux CW, McIver L, Mingrone G, Nece P, Reid TJ, Rogers AM, Rosenbaum M, Seeley RJ, Torres AJ, Dixon JB (2020). Joint international consensus statement for ending stigma of obesity. Nat Med.

[10] Shubeck S, Dimick JB, Telem DA (2018). Long-term outcomes following bariatric surgery. JAMA.

[11] Phelan SM (2018). An update on research examining the implications of stigma for access to and utilization of bariatric surgery. Curr Opin Endocrinol Diabetes Obes.

[12] Dolan P, Afaneh C, Symer M, Dakin GF, Pomp A, Yeo H (2019). Assessment of public attitudes toward weight loss surgery in the United States. JAMA Surg.

[13] Hansen B, Dye MH (2016). Damned if you do, damned if you don’t: the stigma of weight loss surgery. Deviant Behav.

[14] Mattingly BA, Stambush MA, Hill AE (2009). Shedding the Pounds but not the Stigma: negative attributions as a function of a target's method of weight loss. J Appl Biobehav Res.

[15] Trainer S, Benjamin T (2017). Elective surgery to save my life: rethinking the "choice" in bariatric surgery. J Adv Nurs.

[16] Vartanian LR, Fardouly J (2013). The stigma of obesity surgery: negative evaluations based on weight loss history. Obes Surg.

[17] Vartanian LR, Fardouly J (2014). Reducing the stigma of bariatric surgery: benefits of providing information about necessary lifestyle changes. Obesity (Silver Spring).

[18] Puhl RM, Heuer CA (2010). Obesity stigma: important considerations for public health. Am J Public Health.

[19] Jung FU, Luck-Sikorski C, Stroh C, Riedel-Heller SG (2018). [Referral behavior of general physicians for patients with obesity]. Chirurg.

[20] Jung FU, Luck-Sikorski C, König HH, Riedel-Heller SG (2016). Stigma and knowledge as determinants of recommendation and referral behavior of general practitioners and internists. Obes Surg.

[21] Cohn I, Raman J, Sui Z (2019). Patient motivations and expectations prior to bariatric surgery: a qualitative systematic review. Obes Rev.

[22] Berg A (2020). Untold stories of living with a bariatric body: long-term experiences of weight-loss surgery. Sociol Health Illn.

[23] Coulman KD, MacKichan F, Blazeby JM, Donovan JL, Owen-Smith A (2020). Patients' experiences of life after bariatric surgery and follow-up care: a qualitative study. BMJ Open.

[24] Maxwell MJ (2019). An interpretative phenomenological analysis investigating uk female experiences of psychosocial adjustment following bariatric surgery. Qual Rep.

[25] Han SY, Agostini G, Brewis AA, Wutich A (2018). Avoiding exercise mediates the effects of internalized and experienced weight stigma on physical activity in the years following bariatric surgery. BMC Obes.

[26] Raves DM, Brewis A, Trainer S, Han SY, Wutich A (2016). Bariatric surgery patients' perceptions of weight-related stigma in healthcare settings impair post-surgery dietary adherence. Front Psychol.

[27] Brewis A, SturtzSreetharan C, Wutich A (2018). Obesity stigma as a globalizing health challenge. Global Health.

[28] Hansson LM, Rasmussen F (2014). Attitudes towards obesity in the Swedish general population: the role of one's own body size, weight satisfaction, and controllability beliefs about obesity. Body Image.

[29] Jung FU, Dietrich A, Stroh C, Riedel-Heller SG, Luck-Sikorski C (2017). Changes in attitudes towards bariatric surgery after 5 years in the German general public. Obes Surg.

[30] Lee PC, Ganguly S, Tan HC, Lim CH, Chan WH, Kovalik JP, Eng A, Tan J, Lim E, Chua J, Tham KW (2019). Attitudes and perceptions of the general public on obesity and its treatment options in Singapore. Obes Res Clin Pract.

[31] Puhl R, Brownell KD (2003). Ways of coping with obesity stigma: review and conceptual analysis. Eat Behav.

[32] Sikorski C, Luppa M, Dame K, Brähler E, Schütz T, Shang E, König HH, Riedel-Heller SG (2013). Attitudes towards bariatric surgery in the general public. Obes Surg.

[33] Puhl RM, Latner JD, O'Brien K, Luedicke J, Danielsdottir S, Forhan M (2015). A multinational examination of weight bias: predictors of anti-fat attitudes across four countries. Int J Obes (Lond).

[34] Crandall CS, D’Anello S, Sakalli N, Lazarus E, Nejtardt GW, Feather NT (2016). An attribution-value model of prejudice: anti-fat attitudes in six nations. Pers Soc Psychol Bull.

[35] Puhl RM, Lessard LM, Pearl RL, Himmelstein MS, Foster GD (2021). International comparisons of weight stigma: addressing a void in the field. Int J Obes (Lond).

[36] O'Keeffe M, Flint SW, Watts K, Rubino F (2020). Knowledge gaps and weight stigma shape attitudes toward obesity. Lancet Diabetes Endocrinol.

[37] Altaf A, Abbas MM (2019). Public perception of bariatric surgery. Saudi Med J.

[38] Teo EY, Lew PS, Foo CS (2012). Public perceptions of obesity and bariatric surgery in Singapore: a pilot study. Singapore Med J.

[39] Fardouly J, Vartanian LR (2012). Changes in weight bias following weight loss: the impact of weight-loss method. Int J Obes (Lond).

[40] Alreshidi FS, Alshammari TM, Alshammari EM, Alshammari AO, Abdulmogith MA, Albayih ZA, Ahmed HG, Hussain G, Fayez S, Alreshidi TM, Alshammari M, Zaid A (2020). Aerobic exercises and its effects on primary dysmenorrhea among women at Hail city, Saudi Arabia. International Journal of Medicine in Developing Countries.

[41] Lund TB, Nielsen ME, Sandøe P (2015). In a class of their own: the Danish public considers obesity less deserving of treatment compared with smoking-related diseases. Eur J Clin Nutr.

[42] Lund TB, Sandøe P, Lassen J (2011). Attitudes to publicly funded obesity treatment and prevention. Obesity (Silver Spring).

[43] Zacharoulis D, Bakalis V, Zachari E, Sioka E, Tsimpida D, Magouliotis D, Tasiopoulou V, Chatedaki C, Tzovaras G (2018). Current knowledge and perception of bariatric surgery among Greek doctors living in Thessaly. Asian J Endosc Surg.

[44] Fan M, Hong J, Cheung PN, Tang S, Zhang J, Hu S, Jiang S, Chen X, Yu S, Gao L, Wang C, Chen W, Yang W (2020). Knowledge and attitudes towards obesity and bariatric surgery in Chinese nurses. Obes Surg.

[45] Conaty EA, Denham W, Haggerty SP, Linn JG, Joehl RJ, Ujiki MB (2020). Primary care physicians' perceptions of bariatric surgery and major barriers to referral. Obes Surg.

[46] Memarian E, Carrasco D, Thulesius H, Calling S (2021). Primary care physicians' knowledge, attitudes and concerns about bariatric surgery and the association with referral patterns: a Swedish survey study. BMC Endocr Disord.

[47] Goffman E (1963). Stigma: Notes on the management of spoiled identity.

[48] Coulman KD, MacKichan F, Blazeby JM, Owen-Smith A (2017). Patient experiences of outcomes of bariatric surgery: a systematic review and qualitative synthesis. Obes Rev.

[49] Doni K, Breuing J, Pieper D (2020). Psychosocial changes of bariatric surgery in patients' everyday life: a scoping review. Obes Surg.

[50] Crandall CS, Martinez R (2016). Culture, ideology, and antifat attitudes. Pers Soc Psychol Bull.

[51] Dovidio Jf, Major B, Crocker J, Heatherton KF, Kleck RE, Hebl MR, Hull JG (2000). The social psychology of stigma.

[52] Rodhain A, Gourmelen A (2018). Obesity: the link between stigma and perceived responsibility. Journal of Marketing Management.

[53] Stangl AL, Earnshaw VA, Logie CH, van Brakel W, Simbayi LC, Barré I, Dovidio JF (2019). The health stigma and discrimination framework: a global, crosscutting framework to inform research, intervention development, and policy on health-related stigmas. BMC Med.

[54] Fetters MD, Curry LA, Creswell JW (2013). Achieving integration in mixed methods designs-principles and practices. Health Serv Res.

[55] Creswell JW, Plano Clark VL (2017). Designing and conducting mixed methods research, third edition.

[56] Fura B, Wang Q (2017). The level of socioeconomic development of EU countries and the state of ISO 14001 certification. Qual Quant.

[57] Thorlby R (2020). International Health Care System Profiles, England. The Commonwealth Fund.

[58] Wammes J, Stadhouders N, Westert G (2020). International Health Care System Profiles, Netherlands. The Commonwealth Fund.

[59] Durand-Zaleski I (2020). International Health Care System Profiles, France. The Commonwealth Fund.

[60] All-Party Parliamentary Group on Obesity (2020). The Future of Obesity Services: A policy paper produced by the All-Party Parliamentary Group on Obesity.

[61] All-Party Parliamentary Group on Obesity (2020). The current landscape of obesity services: A report from the ALL-Party Parliamentary Group on Obesity.

[62] (2010). Zorgstandaard Obesitas. Partnerschap Overgewicht Nederland.

[63] (2019). Feuille de route: Prise en Charge de personnes en situation d'obésite. Santé MdSedl.

[64] (2010). French obesity plan 2010-2013. Le ministère des Affaires sociales et de la Santé.

[65] Van den heede K, Ten Geuzendam B, Dossche D, Janssens S, Louwagie P, Vanderplanken K, Pascale J (2020). Bariatric surgery in Belgium: organisation and payment of care before and after surgery. Belgian Health Care Knowledge Centre.

[66] Tricco AC, Lillie E, Zarin W, O'Brien KK, Colquhoun H, Levac D, Moher D, Peters MDJ, Horsley T, Weeks L, Hempel S, Akl EA, Chang C, McGowan J, Stewart L, Hartling L, Aldcroft A, Wilson MG, Garritty C, Lewin S, Godfrey CM, Macdonald MT, Langlois EV, Soares-Weiser K, Moriarty J, Clifford T, Tunçalp O, Straus SE (2018). PRISMA extension for scoping reviews (prisma-scr): checklist and explanation. Ann Intern Med.

[67] Swan H (2016). A qualitative examination of stigma among formerly incarcerated adults living with HIV. Sage Open.

[68] Berndt AE (2020). Sampling Methods. J Hum Lact.

[69] Gill SL (2020). Qualitative Sampling Methods. J Hum Lact.

[70] Guest G, Bunce A, Johnson L (2006). How Many Interviews Are Enough?. Field Methods.

[71] Edward K, Hii MW, Giandinoto JA, Hennessy J, Thompson L (2018). Personal descriptions of life before and after bariatric surgery from overweight or obese men. Am J Mens Health.

[72] Graham Y, Hayes C, Small PK, Mahawar K, Ling J (2017). Patient experiences of adjusting to life in the first 2 years after bariatric surgery: a qualitative study. Clin Obes.

[73] Newhook JT, Gregory D, Twells L (2015). 'Fat girls' and 'big guys': gendered meanings of weight loss surgery. Sociol Health Illn.

[74] Warholm C, Marie Øien A, Råheim M (2014). The ambivalence of losing weight after bariatric surgery. Int J Qual Stud Health Well-being.

[75] Bacon JG, Scheltema KE, Robinson BE (2001). Fat phobia scale revisited: the short form. Int J Obes Relat Metab Disord.

[76] Brantley PJ, Waldo K, Matthews-Ewald MR, Brock R, Champagne CM, Church T, Harris MN, McKnight T, McKnight M, Myers VH, Ryan DH (2014). Why patients seek bariatric surgery: does insurance coverage matter?. Obes Surg.

[77] Libeton M, Dixon JB, Laurie C, O'Brien PE (2004). Patient motivation for bariatric surgery: characteristics and impact on outcomes. Obes Surg.

[78] Pearl RL, Wadden TA, Walton K, Allison KC, Tronieri JS, Williams NN (2019). Health and appearance: factors motivating the decision to seek bariatric surgery. Surg Obes Relat Dis.

[79] Rattray J, Jones MC (2007). Essential elements of questionnaire design and development. J Clin Nurs.

[80] Luvisaro BM, Menezes JR, Rodrigues CF, Soares AL, Muzi CD, Guimarães RM (2017). Conceptual equivalence of items and semantic equivalence of the Brazilian version of the EORTC QLQ-ELD14 instrument to evaluate the quality of life of elderly people with cancer. Revista Brasileira de Geriatria e Gerontologia.

[81] Taherdoost H (2016). How to design and create an effective survey/questionnaire; a step by step guide. International Journal of Academic Research in Management.

[82] Sample Size Calculator. Raosoft.

[83] Taherdoost H (2017). Determining Sample Size; How to Calculate Survey Sample Size. International Journal of Economics and Management Systems.

[84] Clarke V, Braun V (2016). Thematic analysis. J Posit Psychol.

[85] Braun V, Clarke V (2006). Using thematic analysis in psychology. Qual Res Psychol.

[86] Eldh AC, Årestedt L, Berterö C (2020). Quotations in qualitative studies: reflections on constituents, custom, and purpose. Int J Qual Methods.

[87] Thorne S (2021). On the use and abuse of verbatim quotations in qualitative research reports. Nurse Author Ed.

[88] Hoofnagle CJ, van der Sloot B, Borgesius FZ (2019). The European Union general data protection regulation: what it is and what it means. Information & Communications Technology Law.

[89] Labott S, Johnson TP, Feeny NC, Fendrich M (2016). Evaluating and addressing emotional risks in survey research. Surv Pract.

[90] Hopkins JC, Howes N, Chalmers K, Savovic J, Whale K, Coulman KD, Welbourn R, Whistance RN, Andrews RC, Byrne JP, Mahon D, Blazeby JM, By-Band Trial Management Group (2015). Outcome reporting in bariatric surgery: an in-depth analysis to inform the development of a core outcome set, the BARIACT Study. Obes Rev.

[91] Ricci L, Lanfranchi JB, Lemetayer F, Rotonda C, Guillemin F, Coste J, Spitz E (2019). Qualitative methods used to generate questionnaire items: a systematic review. Qual Health Res.

[92] Bhogal SK, Reddigan JI, Rotstein OD, Cohen A, Glockler D, Tricco AC, Smylie JK, Glazer SA, Pennington J, Conn LG, Jackson TD (2015). Inequity to the utilization of bariatric surgery: a systematic review and meta-analysis. Obes Surg.

[93] Martin M, Beekley A, Kjorstad R, Sebesta J (2010). Socioeconomic disparities in eligibility and access to bariatric surgery: a national population-based analysis. Surg Obes Relat Dis.

[94] Smith HJ, Chen J, Liu X (2008). Language and rigour in qualitative research: problems and principles in analyzing data collected in Mandarin. BMC Med Res Methodol.

