# Supplementary material for: Stigma Toward Bariatric Surgery in the Netherlands, France, and the United Kingdom: Protocol for a Cross-cultural Mixed Methods Study
Source: JMIR Res Protoc. 2022 Apr 28;11(4):e36753. doi: 10.2196/36753 (PMC9100527; doi:10.2196/36753)
Supplement: Multimedia Appendix 1 [file resprot_v11i4e36753_app1.pdf]

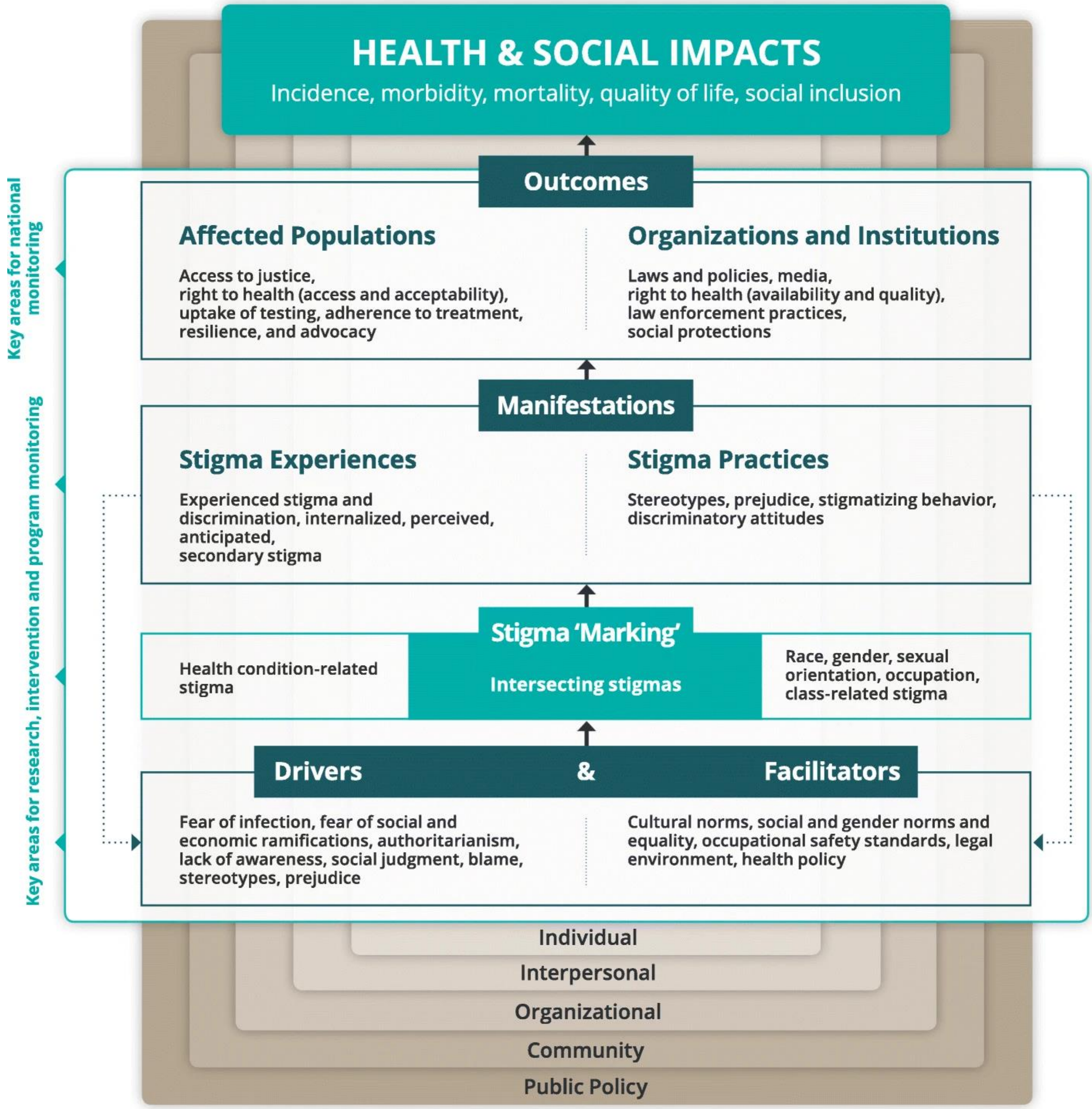

Health Stigma and Discrimination Framework. Reprinted from "The Health Stigma and Discrimination Framework: a global, crosscutting framework to inform research, intervention development, and policy on health-related stigmas," by A. L. Stangl, V. A. Earnshaw, C. H. Logie, W. van Berkel, L. C. Simbayi, I. Barré, and J. F. Dovido, 2019, *BMC Medicine*, 17(31), Copyright (2019) by the Author(s)
