# Supplementary material for: Stigma Toward Bariatric Surgery in the Netherlands, France, and the United Kingdom: Protocol for a Cross-cultural Mixed Methods Study
Source: JMIR Res Protoc. 2022 Apr 28;11(4):e36753. doi: 10.2196/36753 (PMC9100527; doi:10.2196/36753)
Supplement: Multimedia Appendix 2 [file resprot_v11i4e36753_app2.pdf]

Wageningen School of Social Sciences

POSTAL ADDRESS
P.O. Box 8130
6700 EW
The Netherlands

VISITORS' ADDRESS
Building 201
Hollandseweg 1
6706 KN

INTERNET
www.wur.eu/wass
www.wur.nl

Review of Open Round 2019 PhD project "Stigma towards bariatric surgery: Prevalence and impact in the Netherlands, France, and Germany"

[FG4]

Please, rate each aspect on a four-point scale between outstanding and poor by ticking the appropriate box. Please also explain your evaluation.

**Scientific quality**	**Outstanding**	**Good**	**Fair/some weaknesses**	**Poor**
Clarity of the proposal	☐	☒	☐	☐
Originality of the proposal	☒	☐	☐	☐
Use of existing knowledge	☐	☒	☐	☐
Methodological approach	☐	☒	☐	☐
Scientific relevance	☒	☐	☐	☐
Societal relevance	☒	☐	☐	☐

Please, provide detailed comments in support of the rating you have given above; please, also add comments that may help the applicant to improve the proposal if necessary

Comments:
I think that this research is very relevant! Obesity is a public health problem and currently bariatric surgery it the only effective treatment for a large group of patients. From my own perspective I can say that healthcare professsionals can be very negative about BS. I would be great to have more knowledge about how and why these proseffionals, but also other groups have these negative thoughts. What I miss in the proposal is the connection between stigmatisation and treatment outcome. This is of course not the goal of this research, but it is a very important relationship what makes this research even more relevant.
Just a note, the proposal is about obesity stigma, it would be more appropriate to name obese individuals people living with obesity. Practice what you preach.

POSTAL ADDRESS
P.O. Box 8130
6700 EW
The Netherlands

VISITORS' ADDRESS
Building 201
Hollandseweg 1
6706 KN

INTERNET
www.wur.eu/wass
www.wur.nl

Wageningen School of Social Sciences

**Feasibility**	**Outstanding**	**Good**	**Fair/some weaknesses**	**Poor**
Feasibility of the work plan	☒	☐	☐	☐
Appropriateness of the scope of the research	☐	☒	☐	☐
Expertise in the supervision team	☐	☒	☐	☐
Cooperation with others	☒	☐	☐	☐

Please, provide detailed comments in support of the rating you have given above; please, also add comments that may help the applicant to improve the proposal if necessary

Comments:
I would say that it would be more informative to start with the interviews and then base the questionnaires on the interviews. That way you will have more information and more points of view, which you might miss when starting with questionnaires.
The project seems very feasible.

**Collaboration between chair groups**	**Outstanding**	**Good**	**Fair/some weaknesses**	**Poor**
Explanation of the contribution of expertise of each participating group	☐	☒	☐	☐
Strategic importance of the collaboration	☒	☐	☐	☐

Please, provide detailed comments that may help the applicant to improve the proposal if necessary

Comments:
The two groups seem to really complement each other in knowledge and I think thereby collaboration will improve different aspect of the project. What I miss here is more explanation of how these group will collaborate, making sure that the central goal will be achieved.

Thank you very much for you co-operation and please return within two weeks to Wageningen School of Social Sciences: e-mail to: wass@wur.nl

Rebuttal

Stigma towards bariatric surgery: Prevalence and impact in the Netherlands, France, and Germany

Dear members of the WASS committee,

I would like to thank the reviewer for taking the time to review my proposal and for providing constructive feedback. In regard to the terminology used, I agree that we should use an appropriate term when referring to people living with obesity, and therefore will use the term as suggested whenever possible. Below I address additional points discussed in the review.

Connection between stigmatization and treatment outcomes

The reviewer indicates that the relevance of the proposed research could be further strengthened by incorporating the connection between stigmatization and treatment outcomes in the research. I agree with the reviewer that treatment outcome is a very important aspect of stigmatization, one that requires, and will gain, considerable attention in the proposed research. Below I will outline the current evidence on the (unfavourable) relationship between stigmatization and patient outcome for Bariatric Surgery (BS).

The clinical effectiveness of BS is often measured in terms of achieving significant weight loss and the improvement of obesity-related disease (Hopkins et al., 2015). In order to facilitate weight loss and prevent weight regain it is essential for individuals to adhere to a strict diet and exercise regime post-surgery (one that requires considerable effort). Bariatric patients who experience stigma post-surgery are less likely to adhere to these dietary and exercise guidelines (Raves et al. 2016; Hans et al. 2018).

Currently most studies investigating the impact of stigma on BS (indirect) outcomes are quantitative in nature and those of qualitative nature have mostly taken patients perspective into account. To my knowledge, only one mixed method study exists investigating the impact of stigma on patient outcomes from both the patients and health care professional's perspective (Raves et al. 2016). This study, focusing primarily on dietary adherence, showed that BS-patients and health care professionals think differently about the impact of stigma on treatment outcomes. Patients felt that stigma influenced outcomes and health care professionals thought the reverse relationship to be more accurate (Raves et al. 2016). To fully understand how stigma is constructed and to fully capture the impact of stigma on bariatric surgery treatment outcomes more research is needed, not only from the perceptive of patients but also from the perspective of health care professional. The proposed study will address this research gap.

Qualitative data as input for survey study.

The reviewer suggests that it would be more informative to start the project with participant interviews and based the surveys on these interviews. As a result, this would provide more points of view than when starting off with the questionnaire. I would like to thank the reviewer for this helpful suggestion.

To ensure that the questionnaire fully reflects the perspective of people living with obesity and BS patients I have now decided to indeed first conduct the semi-structured interviews. The results of these interviews (together with the results of a literature review) will be used for questionnaire item generation. This way of developing a questionnaire is, according to experts, the most appropriate way of ensuring content validity, a fundamental element of questionnaire validity (Ricci et al., 2019).

Collaboration between chair groups

The reviewer states that the two groups involved in the project complement each other in knowledge and that collaboration will benefit the project. However, the reviewer expresses that there is limited information on how these two groups will collaborate to ensure the goal of the project is achieved. Below I will try to elaborate on the foreseen collaboration.

Both chair groups will be involved in all phases the research project as both groups have extensive general expertise with research projects of a multi-, inter-, or transdisciplinary nature and with mixed-methods studies. Specific expertise of the chair groups will be used for all research phases (including, research set-up, selecting appropriated comparative design and methods, data analysis, & dissemination). For example, HSO and RSO use different ontological perspectives and theoretical perspectives to analyses social (health) issues. While HSO has extensive knowledge on research focused on understanding and solving society relevant challenges and complex health issues from a social-ecological perspective, RSO has extensive knowledge on and experience with conducting international (geographical) comparative studies within Europe (but also with countries such as Ecuador, India, and Turkey). This knowledge and experience will be used to collect and analyse data from France, Germany and the Netherlands. Throughout the project regular meetings and discussions (approximately once a month) on progress and next steps will take place, this way researcher can share content, perspectives, and insights and learn from each other. Prof. dr. Maria Koelen will be the promotor of the project and Prof. dr.ir Hans Wiskerke the co-promotor.

Given that the project involves a set of complex societal questions that cross disciplines (and countries), an integrated research collaboration between both chair groups is essential to achieve the goals of the research project.

I hope that this rebuttal letter addresses the comments by the reviewer sufficiently.

Yours Sincerely,
Franshelis Garcia

References

Han, S., Agostini, G., Brewis, A. A., & Wutich, A. (2018). Avoiding exercise mediates the effects of internalized and experienced weight stigma on physical activity in the years following bariatric surgery. BMC Obesity, 5, 1-9.

Hopkins, J.C., Howes, N., Chalmers., K., Savovic, J., Whale, K., Coulman, K.D., et al. (2015). Outcome reporting in bariatric surgery: An in-depth analysis to inform the development of a core outcome set, the BARIACT study. *Obesity Reviews*, 16, 88–106.

Raves, D.M., Brewis, A., Trainer, S., Han, S, Wutich, A. (2016). Bariatric surgery patients' perceptions of weight-related stigma in healthcare settings impair post-surgery dietary adherence. *Frontiers in Psychology, 7,* 1497.

Ricci, L., Lanfranchi, J.B., Lemetayer, F., Rotonda, C., Guillemin, F., Coste, J., & Spitz, E. (2019). Qualitative Methods Used to Generate Questionnaire Items: A Systematic Review. Qualitativ*, Health Research, 29,* 149–156.
